# An assessment of the test**–**retest reliability of the New Nordic Diet score

**DOI:** 10.3402/fnr.v59.28397

**Published:** 2015-08-11

**Authors:** Helga Birgit Bjørnarå, Elisabet Rudjord Hillesund, Monica Klungland Torstveit, Tonje Holte Stea, Nina Cecilie Øverby, Elling Bere

**Affiliations:** Department of Public Health, Sport and Nutrition, Faculty of Health and Sport Sciences, University of Agder, Kristiansand, Norway

**Keywords:** New Nordic Diet, health, dietary pattern, adherence, diet score, test–retest reliability

## Abstract

**Background:**

There is a growing interest in the New Nordic Diet (NND) as a potentially health promoting, environmentally friendly, and palatable regional diet. Also, dietary scores are gaining ground as a complementary approach for examining relations between dietary patterns and various health outcomes. A score assessing adherence to the NND has earlier been published, yet not tested for reliability.

**Objective:**

To assess the test–retest reliability of the NND score in a sample of parents of toddlers, residing in Southern Norway.

**Design:**

A questionnaire survey was completed on two occasions, approximately 14 days apart, by 67 parents of toddlers [85% females, mean age 34 years (SD=5.3 years)]. The NND score was constructed from 24 items and comprised 10 subscales that summarize meal pattern and intake of typical Nordic foods. Each subscale was dichotomized by the median and assigned values of ‘0’ or ‘1’. Adding the subscales yielded a score ranging from 0 to 10, which was further trichotomized. Test–retest reliability of the final NND score and individual subscales was assessed by Pearson's correlation coefficient and Spearman's rank correlation coefficient, respectively. Additionally, cross tabulation and kappa measure of agreement (*k*) were used to assess the test–retest agreement of classification into the NND score, and the subscales.

**Results:**

Test–retest correlations of the NND score and subscales were *r*=0.80 (Pearson) and *r*=0.54–0.84 (Spearman), respectively, all *p*<0.001. There were 69% (*k*=0.52) and 67–88% (*k*=0.32–0.76) test–retest correct classification of the trichotomized score and the dichotomized subscales, respectively.

**Conclusion:**

The NND score and the 10 subscales appear to have acceptable test–retest reliability when tested in a sample of parents of toddlers.

During the last decades, numerous studies have highlighted associations between adherence to a Mediterranean dietary pattern and health status (1). Despite broad promotion, adherence to this diet is still low outside its traditional geographic regions (2). Suggested barriers for adherence are limited access to ingredients, cultural differences in taste and preferences, and the general difficulty of changing established dietary patterns (3–5). Thus, there is at present a growing interest in whether other regional diets could provide similar health benefits.

The New Nordic Diet (NND) has been proposed as an example of a palatable regional diet, potentially promoting health, environmental sustainability, and preservation of cultural diversity in eating habits (6). The concept NND consists of healthy foods native to the Nordic climate or foods that can be produced in the Nordic climate, such as whole grains, root vegetables, cabbages, berries, certain fruits, wild fish and game, potatoes, and rapeseed oil (6, 7). Intervention studies have reported that adherence to a designed Nordic diet is inversely associated with several cardiovascular risk factors (8), inflammatory markers, and serum lipids (9), as well as positively associated with greater weight loss, blood pressure reduction (10), less body weight regain, and higher dietary satisfaction (11), in at-risk populations. Observational studies have shown that adherence to dietary patterns comprising selected aspects of the Nordic diet is associated with lower total mortality (12), reduced risk of colorectal cancer (13), lower abdominal obesity (14, 15), less body fat (15), and reduced obesity-related markers of inflammation (16). Adherence to the NND has also been associated with optimal gestational weight gain during pregnancy (17), improved fetal growth (17), and lower risk of preeclampsia and spontaneous preterm delivery (18).

Dietary pattern analysis has emerged as a complementary approach for examining the relationship between diet and health status, entailing conceptual and methodological advantages, for example capturing a larger part of overall diet complexity and potential synergistic effects of foods eaten in combination (19–21). Overall, diet is summarized by a single index or score resulting from the combination of included food components. Roughly, score components are selected either *a priori*, based on previous knowledge or scientific evidence, or *a posteriori* using data-driven statistical techniques like factor analysis or cluster analysis (22). Several dietary scores have been constructed for measuring adherence to predefined healthy diets, often evidence-based dietary guidelines (23), whereas others are developed in order to assess compliance with specific regional diets (12, 14, 17, 24). The NND score was constructed *a priori* in order to explore associations between NND adherence with various pregnancy-related health outcomes in women participating in the Norwegian Mother and Child Cohort Study (MoBa) (17, 18, 25). However, there is a lack of studies examining the reliability of such scores (20, 22). Previous studies have assessed the reliability of *a posteriori* derived dietary patterns among adults (26–30), or *a priori* among children (31). Thus, the purpose of the present study was to assess the test–retest reliability of the NND score in a sample of parents of toddlers, residing in Southern Norway.

## Methods

### Design and study sample

An appropriate method for assessing longer-term, habitual dietary intake is the food frequency questionnaire (FFQ), as it is rather inexpensive, can be implemented on a large scale, and implies a modest burden on study participants (32). In the current study, data are derived from the project Healthy and Sustainable Lifestyle (HSL), which in 2014 collected data in collaboration with the Child Food Courage project (33). As part of these projects, an electronic questionnaire was developed for assessing lifestyle behaviors, self-perceived health and life quality among parents of toddlers, and food and eating behaviors among their children. A convenience sample, consisting of parents with children born between 2008 and 2011, was recruited through kindergartens. Parents were informed about the purpose and implications of the study by email and through a web page. For each child, either the mother or the father could participate. In total 1,191 parents from 19 kindergartens in the county of Vest-Agder, Southern Norway, were invited to participate, and 86 parents signed up. Parents provided consent electronically, followed by distribution of the questionnaire survey by email. The time period between the test and the retest distribution was approximately 14 days. In total 75 parents completed the first survey and 67 parents completed the questionnaire at both occasions.

### The NND score

The electronic questionnaire incorporated a FFQ assessing participants’ habitual intake of selected foods, among them typical Nordic foods. Only frequency of consumption was assessed, the items did not specify portion sizes or amount. The NND score was previously constructed in order to capture adherence to the concept of the NND (17), where health, sustainability, gastronomic potential, and Nordic identity are fundamental principles (34); and it comprises 10 subscales summarizing meal pattern and intake of typical Nordic foods. Table 1 describes the components underlying the construction of the 10 subscales, including related questionnaire items and response options. Meal pattern was included in the score due to the potentially favorable impact of routine consumption of meals on dietary quality (35, 36). Furthermore, meat from game (moose, reindeer, deer), wild fish, other seafood, and berries were collapsed into one subscale (‘Foods from the wild countryside’), as these foods are characterized by a common reliance on soil and local vegetation (17). Also, such a combination of foods is in line with one of the specific guidelines of the concept NND: ‘More foods from the wild countryside’ (34). In the present study, the number of indicator questions for the subscales ranged from 1 to 5, in total 24 questions. Question formulation was as follows: ‘How often do you eat…’, or ‘How often do you drink…’, with 10 response options ranging from ‘Never’ (coded 0), up to ‘Several times a day’ (coded 10). Each subscale was dichotomized by the median and assigned values of ‘0’ or ‘1’, with ‘1’ indicating a more frequent consumption of main meals (subscale 1), or a more favorable intake of the relevant foods (subscale 2–10). Adding the subscales yielded a score ranging from 0 to 10, implying that each subscale was given equal weighting. Increasing score expressed higher compliance with the NND. This procedure is in line with methods applied in previous studies exploring relations between adherence to the Mediterranean diet (24) and selected healthy aspects of the Nordic diet (12) with health parameters. The score was further trichotomized, grouping participants into ‘low’ (0–3 points), ‘medium’ (4–5 points), and ‘high’ (6–10 points) adherence to the NND. The cut-offs were determined to obtain the most equally sized groups.

**Table 1:** The components underlying the construction of the subscales within the NND score (*n=*67)

Subscale		Related question(s)	Response alternatives and coding	Calculations (min–max)	Median=cut-off	Dietary behavior associated with scoring
1:	Meal pattern	How often do you eat breakfast lunch dinner evening meal/supper	Never=0 Less than once a week=0.5 Once a week=1 Twice a week=2 Three times a week=3 Four times a week=4 Five times a week=5 Six times a week=6 Every day=7	Sum of answers to the four questions (0–28)	Test: 24.0 Retest: 24.0	Test: ≤24.0=0 ≥25.0=1 Retest: ≤24.0=0 ≥25.0=1
2:	Nordic fruits	How often do you eat typical Nordic fruits (apple, pear, plum)	Never=0 Less than once a week=0.5 Once a week=1 Twice a week=2 Three times a week=3 Four times a week=4 Five times a week=5 Six times a week=6 Every day=7 Several times a day=10	No calculation (0–10)	Test: 4.0 Retest: 4.0	Test: ≤4.0=0 ≥5.0=1 Retest: ≤4.0=0 ≥5.0=1
3:	Root vegetables	How often do you eat root vegetables (e.g. carrot, rutabaga, onion)?	Never=0 up to Several times a day=10	No calculation (0–10)	Test: 5.0 Retest: 4.0	Test: ≤5.0=0 ≥6.0=1 Retest: ≤4.0=0 ≥5.0=1
4:	Cabbages	How often do you eat cabbages (e.g. cauliflower, broccoli, brussel sprouts, kale)?	Never=0 up to Several times a day=10	No calculation (0–10)	Test: 3.0 Retest: 3.0	Test: ≤3.0=0 ≥4.0=1 Retest: ≤3.0=0 ≥4.0=1
5:	Potatoes vs. rice/pasta	How often do you eat potatoes rice pasta	Never=0 up to Several times a day=10	Frequency of eating potatoes relative to eating rice and pasta combined: potatoes/(rice+pasta) (0–100)	Test: 0.49 Retest: 0.49	Test: <0.49=0 ≥0.49=1 Retest: <0.49=0 ≥0.49=1
6:	Whole grain breads vs. white breads	How often do you eat white breads/bread rolls whole grain breads whole grain hard breads	Never=0 up to Several times a day=10	Frequency of eating whole grain breads and hard breads combined relative to eating refined breads: (whole grain breads+whole grain hard breads)/refined breads (0–200)	Test: 14.67 Retest: 12.00	Test: ≤14.67=0 >14.67=1 Retest: ≤12.0=0 >12.0=1
7:	Oatmeal porridge	How often do you eat oatmeal porridge?	Never=0 up to Several times a day=10	No calculation (0–10)	Test: 1.0 Retest: 0.5	Test: <1.0=0 ≥1.0=1 Retest: ≤0.5=0 >0.5=1
8:	Foods from the wild countryside	How often do you eat game (e.g. moose, reindeer, deer) lean fish (e.g. cod, caley, haddock) fatty fish (e.g. mackerel, herring, halibut) other seafood (e.g. shrimps, crabs, mussels) berries	Never=0 up to Several times a day=10	Sum of answers to the five questions (0–50)	Test: 4.5 Retest: 4.5	Test: ≤4.5=0 ≥5.0=1 Retest: ≤4.5=0 ≥5.0=1
9:	Milk vs. juice	How often do you drink milk fruit juice without added sugar	Never=0 up to Several times a day=10	Frequency of drinking milk relative to drinking fruit juice: milk/juice (0–100)	Test: 1.37 Retest: 0.99	Test: ≤1.37=0 >1.37=1 Retest: ≤0.99=0 >0.99=1
10:	Water vs. sugar/artificially sweetened beverages	How often do you drink water sugar sweetened beverages artificially sweetened beverages	Never=0 up to Several times a day=10	Frequency of drinking water relative to drinking sugar sweetened beverages and artificially sweetened beverages combined: water/(sugar sweetened beverages+artificially sweetened beverages) (0–100)	Test: 4.76 Retest: 4.38	Test: ≤4.76=0 >4.76=1 Retest: <4.38=0 ≥4.38=1

NND, New Nordic Diet.

### Statistical analysis

Statistical analyses were performed using the statistical software package IBM SPSS Statistics version 22.0 (IBM Corp., Somers, NY, USA). Test–retest reliability of the subscales and the final NND score was investigated through bivariate correlations. As the distributions of the subscales were skewed, correlations were computed with Spearman's rank correlation coefficient, whereas the final NND score was presented with Pearson's correlation coefficient, due to a normal distribution of scores. Furthermore, cross tabulation and kappa measure of agreement (*k*) were applied for assessing the test–retest agreement of classification into the trichotomized NND score, as well as into the dichotomized subscales. A two-sided *p*-value of <0.05 was considered statistically significant.

## Results

The questionnaire survey was completed on both occasions by 67 participants (89% of those answering the first questionnaire), mean age 34.5 years (SD=5.3). In total 57 participants (85%) were females, 60 participants (90%) were native Norwegians, and 36 participants (54%) reported 4 years or more of university or college education. Table 2 presents details for the results from the test–retest analyses. The correlation coefficients between test and retest were *r*=0.80 (Pearson) for the NND score, and *r*=0.54–0.84 (Spearman's rank correlation coefficient) for the different subscale scores, all *p*<0.001. The lowest correlation was seen for the subscale ‘cabbages’ (*r*=0.54), whereas the highest correlations were observed for the subscales ‘oatmeal porridge’ and ‘milk vs. juice’ (*r*=0.84). Regarding the test–retest agreement of the trichotomized NND score, 69% of participants were correctly classified into low, medium, or high adherence on the second occasion, compared with the first one (*k*=0.50), whereas 1.5% (*n=*1) were grossly misclassified, moving from high to low compliance. For the dichotomized subscales, test–retest correct classification ranged from 67 to 88% (*k*=0.32–0.76). In line with the results from the bivariate correlations, the lowest agreement from test to retest was observed for the subscale ‘cabbages’ (67%, *k*=0.32), whereas the highest agreement was detected for ‘milk vs. juice’ (88%, *k*=0.76).

**Table 2:** Test–retest reliability of the 10 subscales and of the total NND score (*n=*67)

The 10 subscales constituting the NND score		Spearman's rank order correlation	Kappa measure of agreement (dichotomized subscales)	Percent agreement between test and retest (dichotomized subscales)
1:	Meal pattern	0.78	0.70	85
2:	Nordic fruits	0.76	0.60	81
3:	Root vegetables	0.71	0.63	82
4:	Cabbages	0.54	0.32	67
5:	Potatoes vs. rice/pasta	0.70	0.67	84
6:	Whole grain breads vs. white breads	0.62	0.52	76
7:	Oatmeal porridge	0.84	0.67	84
8:	Foods from the wild countryside	0.70	0.51	76
9:	Milk vs. juice	0.84	0.76	88
10:	Water vs. sugar/artificially sweetened beverages	0.79	0.43	72
NND score		0.80^a^	0.52^b^	69^b^

NND, New Nordic Diet. *P*-values for all analyses were <0.001.

a Pearson correlation coefficient is used for the NND score.

b Trichotomized score.

## Discussion

In the present study, we found acceptable test–retest reliability of the previously developed NND score (17). The test–retest correlation coefficients for the subscales ranged from 0.54 to 0.84, while the test–retest correlation for the total NND score was 0.80, all highly significant. This result can be considered acceptable, as correlation coefficients in the order of 0.50 to 0.70 appear typical for reproducibility of nutrient intakes, and is comparable with that of several biological measurements in subjects under real-life conditions (32). In the context of previous studies, Hu et al. (26) assessed the test–retest reliability of two dietary patterns (the ‘prudent’ and ‘western’) defined by factor analysis, based on dietary data from a FFQ administered twice 1 year apart, in a subsample of 127 men from the Health Professionals Follow-up Study. This latter mentioned study, reported correlation coefficients from test to retest ranging from 0.36 to 0.92 for the individual foods, 0.70 for the ‘prudent’ pattern, and 0.67 for the ‘western’ pattern. Using the same dietary data as the study by Hu et al. (26), Newby et al. (27) computed two Dietary Quality Index Revised (DQI-R) scores, and reported the reliability correlation (Pearson) for the two FFQ scores to be 0.72. Furthermore, Khani et al. (28) defined three dietary patterns using factor analysis on data derived from a FFQ, also completed twice 1 year apart, in a subsample of 212 women participating in the Swedish Mammography Cohort. In this study, Spearman correlation coefficients for the patterns ‘healthy’, ‘western’, and ‘drinker’ were reported to be 0.63, 0.68, and 0.73, respectively. In a sample of Japanese men (*n=*244) and women (*n=*254), Nanri et al. (29) explored test–retest reliability of three Japanese dietary patterns (the ‘prudent’, ‘westernized’, and ‘traditional’, identified by principal component analysis) and found that Spearman correlation coefficients ranged from 0.55 to 0.77. Although not entirely comparable due to methodological differences (such as *a posteriori* defined patterns, 1 year instead of approximately 2 weeks between questionnaire administrations, and larger samples), these correlation coefficients are somewhat lower than the ones presented in our study. One possible explanation could be the time interval between administrations. A time period of 1 year may reduce the reproducibility as a result of true changes in dietary intake, as well as variation in response, and not necessarily express poor questionnaire performance (32).

In addition to performing bivariate correlation analyses for exploring test–retest reliability, we applied kappa measure of agreement, combined with observed percentage agreement, as a measure of chance-corrected proportional agreement. According to Altman (37), values of kappa above 0.80 express very good agreement, 0.61–0.80 good agreement, 0.41–0.60 moderate agreement, 0.21–0.40 fair agreement, and <0.20 poor agreement. Thus, 67–88% correct classification of the subscales from test to retest, and kappa measures of agreement of *k*=0.32–0.76, suggests acceptable test–retest reliability. Regarding the total NND score, 69% correct classification, a kappa value of 0.52, and less than 2% grossly misclassified, supports the indication of an acceptable test–retest reliability (38). For comparison, Beck et al. (30) investigated the reliability of iron-related dietary patterns, derived from an FFQ administered twice, 4 weeks apart, in a convenience sample of 115 young women, applying correlation coefficients, cross-classification, and weighted kappa (*k* ^*w*^). Beck and colleagues reported correlations from test to retest to be 0.76 for both dietary patterns identified, the ‘healthy’ and ‘sandwich and drinks’, whereas 63% (*k* ^*w*^=0.57) and 71% (*k* ^*w*^=0.65) were correctly classified into the same tertile, and less than 2% were grossly misclassified, into the ‘healthy’ or ‘sandwich and drinks’ patterns, respectively. Furthermore, Huybrechts et al. (31) tested the reliability of a diet quality index for children, assessed with an FFQ filled in twice, 5 weeks apart, by parents of 58 preschoolers. This study reported Pearson correlation to be 0.88 from test to retest; 62% of the subjects were correctly classified from test to retest, and 3% were classified in extreme categories (31). These two latter studies present results much in line with our findings, yet direct comparisons should be made with caution because of different methodological approaches. However, considering the time period between questionnaire administrations, the study of Beck et al. (30), as well as the study of Huybrechts et al. (31), were relatively comparable to our study. Although a definite answer to an ideal time interval may not exist, a time period as long as 1 year could disrupt evaluation of true questionnaire performance (32).

Regarding the subscales in the present study, 4 out of 10 were based on one questionnaire item only, providing few response alternatives and hence a skewed distribution. Consequently, the dichotomization by the median resulted in slightly different sized groups for some subscales. Still, considering previous study results (17, 18), we feel confident that the method is sufficient for ranking and segregating participants according to adherence to the NND. Besides, the total NND score, which was the main outcome in the present study, was normally distributed. Another study limitation is that neither the questionnaire, from which the NND score is derived, nor the score itself, has been validated. However, regarding FFQs, validity studies are generally difficult to carry out because of the lack of a perfect standard reference method (32), and difficulties of obtaining sufficiently large and representative samples of the population to which the FFQ may be applied. In addition, the NND score inquires dietary behavior rather than absolute intake, making validation even more challenging. Although quantification of foods in the questionnaire probably would result in greater accuracy, it would also increase participant burden.

In terms of the study sample, number of participants is a limitation because approximately 100 subjects, as used in other studies, would have been preferable (32, 39, 40). Moreover, the generalizability is limited due to the low response rate, and further characteristics of the parents who signed up, the majority of whom were female, ethnic Norwegian, and higher educated. Also, because the participants were relatively young and well-educated parents of small children, they could be more motivated than other populations regarding diet, nutrition, and health issues in general, which may result in reliable and repeatable answers, and thus an overestimation of the true reliability of the NND score. Considering previous study results (30, 31), and the general difficulties of measuring dietary intake (32), we believe that the misclassification of 31% of the participants from test to retest reflects the sources of error that are likely to be an inevitable part of dietary research. Nevertheless, such errors represent limitations that need to be taken into account when interpreting study results. The aforementioned characteristics of our study sample may entail that the sources of error could be more pronounced than what we have captured in the present study. Regarding the time interval between the test and the retest administrations of the questionnaire, 2 weeks is relatively short, implying that the participants might remember what they answered in the first questionnaire, which in turn would increase reliability due to memory, and not necessarily as a result of questionnaire performance. Nevertheless, a great range of different time intervals between administrations has been used in previous studies (41). It should also be mentioned that not all foods typical for the NND are included in the score, for example, nuts and seeds, legumes, rapeseed oil, free-range livestock, fresh herbs, and wild plants and mushrooms (34), because of some limitations of the availability of food data. However, the score comprised most food items captured by the concept of NND.

## Conclusion

Based on the acceptable test–retest reliability of the total NND score and its subscales revealed in the present study, together with previous study results, we believe that the NND score is qualified for ranking and segregating subjects according to degree of adherence with the NND, and for detecting potential associations between degree of compliance with various health outcomes. Yet, the reliability of the NND score should be tested in a larger sample and among different subgroups of the Nordic population.
